# Reply to comment on Fisichella et al. (2012), “Intestinal toxicity evaluation of TiO_2_ degraded surface-treated nanoparticles: a combined physico-chemical and toxicogenomics approach in Caco-2 cells” by Faust et al.

**DOI:** 10.1186/1743-8977-9-39

**Published:** 2012-11-01

**Authors:** Matthieu Fisichella, Frédéric Bérenguer, Gérard Steinmetz, Mélanie Auffan, Jérôme Rose, Odette Prat

**Affiliations:** 1 CEA, IBEB, SBTN, LEPC, Bagnols-sur-Cèze, F-30207, France; 2 CEREGE, UMR 7330 CNRS/ Aix-Marseille Université, ECCOREV, Europôle de l’Arbois, Aix-en-Provence, F-13545, France; 3 International Consortium for the Environmental Implications of Nanotechnology (iCEINT), Aix-en-Provence, France

## Abstract

In this response, we discuss the major differences that clearly distinguish our results from those mentioned by Faust et al. In particular, the experiments have been conducted on nanoparticles of different nature, what mainly explains the observed discrepancies. This is a reply to http://www.particleandfibretoxicology.com/content/pdf/1743-8977-9-39.pdf.

## Background

We thank our colleagues, Faust et al. for reading our manuscript that was published in Particle and Fibre Toxicology recently [1] and pointing out differences relative to their own publication [2]. In their letter, Faust et al. indicated that: “The Fisichella et al. study employed surface-treated TiO_2_ nanoparticles (NPs) … Although these conflicting data may be a result of the nanomaterials employed…”

We claim that this is not just a slight discrepancy, but the key difference between these two studies; indeed we do not use the same nanoparticles. The various physicochemical features of nanoparticles are now well known to lead to different interactions with biological systems and induce various toxicities [3]. We used surface-treated TiO_2_ nanoparticles, whereas Koeneman et al. used unmodified TiO_2_ nanoparticles.

• In their study, Koeneman et al. observed adsorption of unmodified TiO_2_ NPs on Caco-2 cells and they conclude as follows:” Results of this investigation support our hypothesis that TiO_2_, although not toxic, has previously undescribed non-lethal effects on microvilli and intracellular-free calcium”.

• Fisichella et al. do not observe any internalization, nor any cytotoxicity nor genomics effects in Caco-2 cells after exposure to surface-treated TiO_2_ NPs.

In their study, after 24h exposure to 100 μg/ mL of unmodified TiO_2_ NPs, Koeneman et al. observed that 29.6% of TiO_2_ NPs were absorbed on the cell surface, with 0.4% of the cells occupied by NPs (Figure four). Their SEM image (Figure seven) indicated that microvilli are damaged in the presence of 1000 μg/mL of unmodified TiO_2_ Nps, and “no longer stood erect” at 10 μg/mL due to NPs absorption onto cell surface. We considered that our results were consistent with those of Koeneman, regarding the lack of toxicity observed in their study for similar doses (10 μg/mL) of TiO_2_ NPs in identical cells.

In our work, after the ‘environmental’ or ‘acidic’ alteration of the T-liteSF (composed of a nanoTiO_2_ core coated with aluminum hydroxide and PDMS layers), 90% of the organic layer is desorbed while the aluminum hydroxide layer persists at the surface of the nano-TiO_2_ core. Faust et al. invoke a possible degradation of the aluminum layer, seen by others [4] in chlorine for 7 days, but this is not the process we used (3h under acidic conditions to mimic gastric degradation) and the protective aluminum layer remains at the NPs surface in our experimental conditions. This is confirmed by the similar ^27^Al NMR spectra of the T-LiteSF before and after alteration [5] showing that the aluminum hydroxide layer at the surface of the nanoTiO_2_ is intact after alteration. The persistence of this aluminum hydroxide layer is also confirmed by chemical analysis (ICP-AES measurements). We demonstrated [1] the lack of generation of superoxide by the surface-treated TiO_2_ NPs, degraded or not, while unmodified TiO_2_ NPs have photocatalytic activity. The ability of unmodified TiO_2_ NPs to generate superoxides likely explains the adverse effects (very relative, given the high tested concentrations) observed by our colleagues. On the contrary, the protective effect of the remaining aluminum hydroxide layer explains the absence of toxicity described in Fisichella et al.

Faust et al. do not have the same interpretation of our SEM pictures of Caco-2 cells (Figure 5B, Lanes 2 and 3); they observed possible distorted microvilli as a proof of harmful effects of our surface-treated TiO_2_ NPs. However it is important to note that the same figure shows untreated cells (Figure 5B, lane 1) displaying the same features of microvilli. Faust et al. claim that our TEM images: "do not resemble Caco-2 images and show a significant effect of their NP treatment” because “normal Caco-2 epithelium exhibit cells with polarized cytoplasm containing many electrondense organelles and cytoplasmic granules with a well-ordered array of microvilli at the cellular apex”.

The absence of well-ordered microvilli is simply due to the cutting method, which involved a parallel cut to the bottom of the Petri dish in order to collect larger fragments, and not transversally as was done by Koeneman et al. [2]. This is a key point in our method that explains the apparent disorder of the MV in Figure 6. A propos the invisible organelles, the lack of contrast of the TEM image on the right in Figure 6 is due to the strong density of NPs on the cell surface, occulting intracellular details.

To conclude, these two studies do not lead to conflicting data, but are complementary studies, based on different nanoparticles, showing the importance of taking into account the surface properties of the NPs in toxicological studies.

Our colleagues will certainly agree with us that these data underscore the need to further examine the toxicological effects, not only of unmodified nanoparticles (i.e. at the beginning of the nanomaterials lifecycle), but of surface-treated nanoparticles used in engineered nanomaterials. At each stage of the lifecycle, from production to the end of life, the surface properties of the nanoparticles will be different leading to different toxicological effects.

We would like to emphasize the importance of evaluating the toxicity of engineered nanomaterials throughout their life cycle in order to design them more safely in the future.

## Competing interests

The authors declare that they have no competing interests.

## Authors’ contributions

All authors read and approved the final manuscript.
